# A systematic review on oncological outcomes, functional results, and laryngeal preservation in vertical partial laryngectomies vs. transoral laser microsurgery for early stage glottic cancer

**DOI:** 10.1007/s00405-025-09657-6

**Published:** 2025-09-03

**Authors:** Sara Bassani, Rogerio Aparecido Dedivitis, Gabriele Molteni, Erica Zampieri, Cecilia Dalmazzini, Mario Augusto Ferrari de Castro, Luiz Paulo Kowalski

**Affiliations:** 1 Otolaryngology-Head and Neck Surgery Department, University of Verona, Piazzale L.A. Scuro, 10, Verona, 37134 Italy; 2 Department of Head and Neck Surgery, University of Sao Paulo School of Medicine, Sao Paulo, Brazil; 3 Department of Medical and Surgical Sciences, Alma Mater Studiorum, University of Bologna, Bologna, Italy; 4 Otolaryngology and Audiology Unit, IRCCS Azienda Ospedaliero- Universitaria of Bologna, Bologna, Italy

**Keywords:** Transoral laser microsurgery, Vertical partial laryngectomy, Glottic carcinoma, Larynx preservation, Survival, Voice, Swallowing

## Abstract

**Purpose:**

To compare the oncological and functional outcomes of transorallaser microsurgery (TLM) and vertical partial laryngectomy (VPL) in earlystageglottic carcinoma (T1-T2).

**Methods:**

A systematic review following PRISMA guidelines analyzedcomparative studies from 2000 to 2024 in PubMed, EMBASE, and Web ofScience. Outcomes included local control, recurrence rates, larynxpreservation, survival, voice quality, and complications.

**Results:**

Eight studies met inclusion criteria. TLM and VPL showed comparablesurvival rates for T1 tumors, but VPL provided better local control and larynxpreservation in T2 and anterior commissure involvement cases. TLM had higherrecurrence risk but superior functional outcomes, including better voicepreservation, shorter hospital stays, and lower complication rates.

**Conclusion:**

Both techniques are viable, but TLM is preferred for T1 tumors,while VPL should be considered for T2 lesions because of higher local controlrates. Patient priorities and tumor characteristics should guide surgical choice.

## Introduction

Early-stage glottic cancer (T1-T2, N0/Stage I-II) generally boasts a favorable prognosis, with effective treatment options including radiotherapy (RT), open surgery, and transoral laser microsurgery (TLM) [1]. Treatment selection is influenced by multiple factors such as physician and patient preferences, resource availability, potential side effects, and expected functional outcomes [2]. Even though a systematic review highlighted the excellent survival rates associated with open vertical partial laryngectomy (VPL), it did not compare those outcomes with TLM results [3]. Furthermore, another review by Campo et al. [4] focused on glottic T2 tumors undergoing VPL, TLM, and radiotherapy. Interestingly while RT demonstrated the poorest 5-year survival rates, it remains the most commonly employed treatment. Conversely, VPL yields the highest rates of laryngeal preservation and survival; however, it is utilized far less frequently in clinical practice and is progressively declining due to the availability of less invasive techniques, raising important questions regarding treatment paradigms for early-stage glottic cancer [5]. Open surgical techniques remain essential in specific cases, especially when tumors involve the anterior commissure, an area that can be challenging to access using transoral methods. One notable advantage of VPL is the capacity for vocal fold reconstruction, potentially enhancing post-treatment voice quality through techniques such as false cord flaps [6]. 

As the transition from open to endoscopic approaches accelerates, there is a pressing need for updated comparative data on oncological and functional outcomes of these techniques. This review aims to analyze the existing literature to assess potential differences in the outcomes between these two surgical approaches.

## Methods

This systematic review evaluates primary studies comparing oncological and functional outcomes between patients with early stage glottic cancer undergoing TLM and VPL. The research methodology adhered to the recommendations for conducting systematic reviews proposed by the Cochrane Collaboration. The titles and abstracts of comparative studies identified in the electronic search were reviewed. Those that met the criteria were included for evaluation. This systematic review was prospectively registered in the PROSPERO international database for systematic reviews (Registration ID: 1049980).

### Search strategy

We strictly followed the Preferred Reporting Items for Systematic Reviews and Meta-Analyses (PRISMA) [7] guidelines.

For our search strategy, we conducted a comprehensive systematic search for articles published between January 2000 and December 2024 in the PubMed, EMBASE, and Web of Science databases. Our query incorporated the following terms: ((Vertical partial laryngectomy) OR (partial vertical laryngectomy) OR (VPL) OR (vertical laryngectomy) OR (fronto-lateral laryngectomy) OR (frontolateral laryngectomy) OR (hemilaryngectomy) OR (hemi-laryngectomy)) AND ((Endoscopic laser cordectomy) OR (laser cordectomy)) AND (outcomes). After conducting the search, we screened the full texts of relevant studies for final selection. All studies identified by the initial literature search were independently reviewed by two authors (SB and EZ). Both titles and abstracts were assessed, and in cases of uncertainty, the full text was examined. Any disputes were resolved by a senior author (RD, GM). The search results were further refined based on inclusion and exclusion criteria, and duplicate records were removed. Additional relevant studies were identified through manual reference checking of included articles.

This review adheres to the Patient, Intervention, Comparison, Outcome, and Study Design (PICOS) framework to systematically select studies focused on early-stage glottic laryngeal cancer. The interventions analyzed include VPL compared to TLM, encompassing laser cordectomy and laser-assisted resections. Both oncological outcomes and functional results, as well as postoperative complications and larynx preservation, were evaluated to compare the effectiveness and safety of these techniques.

### Inclusion and exclusion criteria

Studies were included in the systematic review if they met the following criteria:

- Population: Patients diagnosed with early-stage glottic laryngeal cancer.
- Intervention: Studies evaluating VPL procedures.
- Comparison: Studies comparing TLM.
- Outcomes: Studies reporting at least one of the following criteria:
  - Oncological outcomes: overall survival (OS), disease-free survival (DFS), local control, recurrence rates.
  - Functional outcomes: voice quality, speech outcomes, swallowing function, respiratory performance, larynx preservation rates.
  - Postoperative complications: surgical site infections, airway stenosis, length of hospital stay.
- Study design: Comparative studies (retrospective or prospective) that directly analyze outcomes between open and laser-assisted laryngectomy.
- Studies published between 2000 and 2024.

Articles were excluded if they met any of the following criteria:

- Non-comparative studies, such as single-arm studies evaluating only one surgical technique.
- Case reports, small case series (< 10 patients), narrative reviews, and meta-analyses.
- Studies lacking oncological, functional, or complication-related outcomes.
- Studies involving multimodal treatments (e.g., surgery combined with chemoradiotherapy) without separate surgical outcome analysis.
- Non-English language publications or studies published before 2000.

### Data collection

For this systematic review, data were systematically extracted and compiled into a structured database that included study details (year of publication, country, first author, and study design) as well as patient characteristics (number of patients, tumor subsite, staging). Surgical interventions were classified as endoscopic procedures or open partial laryngectomies, with the number of patients recorded for each technique. Outcomes included oncological results (overall survival, disease-free survival, local control, recurrence rates) and functional outcomes (length of hospitalization, post-treatment complications, voice and swallowing function, and larynx preservation rates).

### Level of evidence and methodological quality

The methodological quality of the selected studies was meticulously evaluated to assess the strength of their evidence and the validity of their inclusion in this review. The classification of the degree of recommendation, reflecting the scientific rigor of each study, was based on the framework established by the Oxford Centre for Evidence-Based Medicine Levels of Evidence [8]. Table 1 summarizes the methodological characteristics and potential risk of bias of the eight included studies. The overall risk of bias was moderate to high due to the retrospective nature, variable follow-up durations, and inconsistent use of standardized functional outcome measures. Only one study was prospective, providing stronger methodological rigor. Despite these limitations, all studies met the inclusion criteria in terms of clearly defined patient populations and outcomes of interest.

**Table 1:** Risk of bias summary

First author, year	Study design	Level of evidence	Risk of bias	Notes
Luo et al., 2021	Retrospective cohort	3b	Moderate	Balanced groups, clear endpoints
Campo et al., 2021	Systematic review	4	High	Non-comparative, pooled estimates
De Campora et al., 2001	Retrospective cohort	3b	High	Limited methodology reporting
Mantsopoulos et al., 2012	Prospective cohort	2b	Low	Only prospective study; robust data
Karatzanis et al., 2009	Retrospective cohort	3b	Moderate	Clear criteria, no blinding
Sewnaik et al., 2005	Retrospective cohort	3b	Moderate	Focus on post-RT salvage cases
Harada et al., 2015	Retrospective cohort	3b	Moderate	Small sample, selective population
Langer et al., 2024	Retrospective cohort	3b	Low	Recent data, standardized outcomes

## Results

**Fig. 1:**
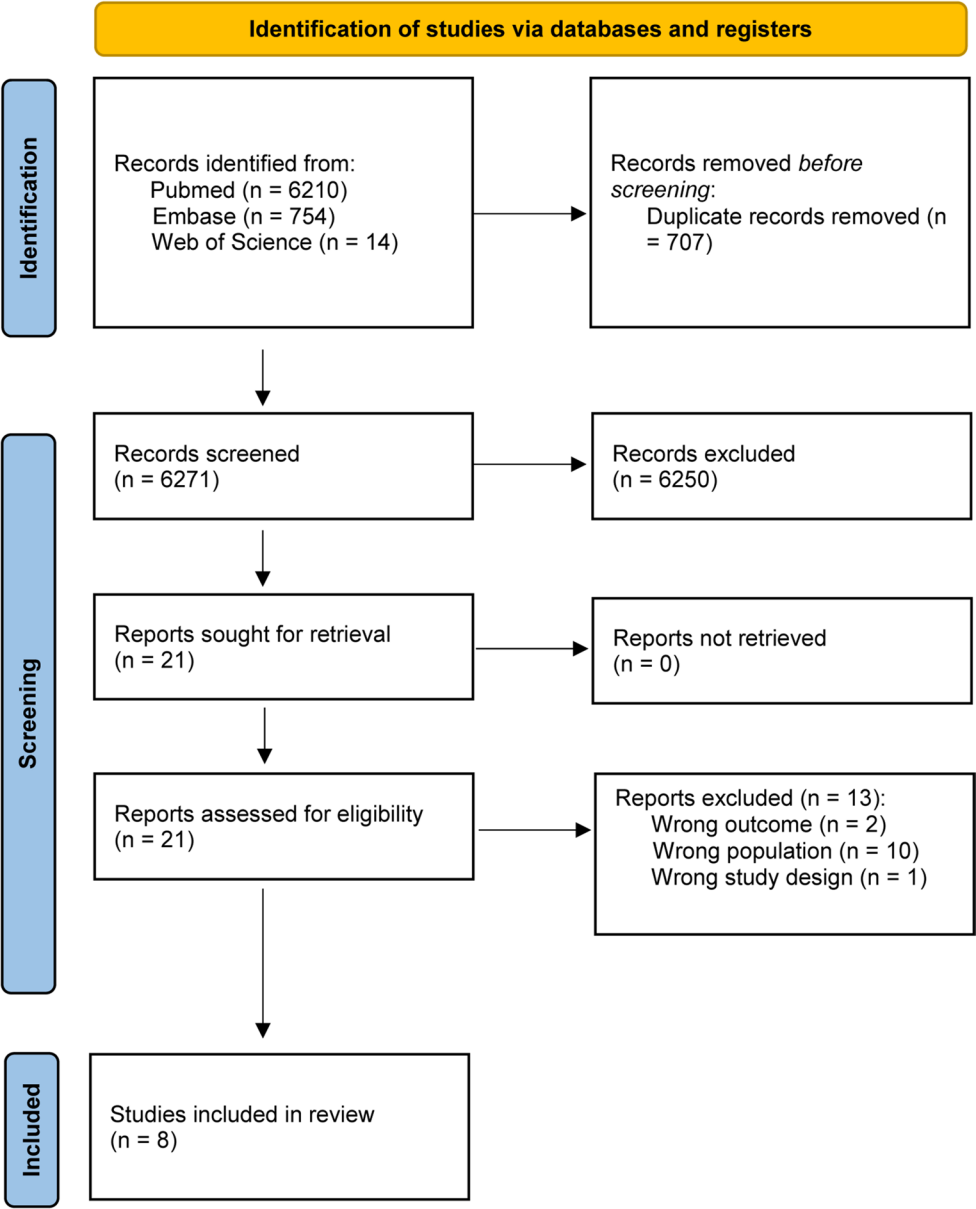
PRISMA. Overview of the articles’ selection process

In the initial phase of the literature review, we systematically examined a total of 6,271 articles, after excluding duplicates. Following a comprehensive evaluation of the titles and abstracts, we identified 21 articles for full-text screening to assess their eligibility. Ultimately, 8 articles met our predefined inclusion criteria (Fig. 1).

This review synthesizes the findings from multiple studies, encompassing patient populations ranging from 63 to 573 individuals, with a mean follow-up period of 24 to 77 months. This comprehensive analysis aims to provide a nuanced understanding of these treatment modalities.

### Oncological outcomes and local control

One of the primary considerations in assessing the efficacy of a surgical approach is its ability to achieve local control (LC) and disease-free survival (DFS). Several studies have demonstrated comparable LC rates between TLM and VPL for T1-stage tumors, suggesting that both techniques are effective when used in appropriately selected cases. Luo et al. [9] reported LC rates at 1, 3, and 5 years of 96.9%, 85.0%, and 85.0%, resepectively for TLM, whereas for VPL, the rates were 98.3%, 95.5%, and 93.2%, respectively. The differences observed between the two techniques were not statistically significant for T1 lesions, suggesting that TLM can be a valid first-line option for these patients​.

In contrast, for T2 tumors, the oncological advantage of VPL becomes more pronounced. Campo et al. [4] reported that VPL provided a superior LC rate of 94.4%, compared to 75.4% for TLM, highlighting the potential limitations of TLM in managing more advanced disease​. Similar findings were reported by De Campora et al. [5]who observed disease-free survival rates of 81.3% for laser cordectomy and 85.7% for VPL, further supporting the notion that VPL may offer more durable oncological outcomes​ (Table 1).

Mantsopoulos et al. [10] and Karatzanis et al. [11] contributed to this discussion by analyzing larger cohorts, finding no statistically significant differences in local control between TLM and VPL, when proper patient selection criteria were applied. These findings suggest that while VPL offers superior control for more deeply infiltrative cancers or those involving the anterior commissure, TLM remains a viable alternative for well-delineated, less extensive lesions (Table 2).

**Table 2:** Oncological outcomes after TLM or VPL

Study	Tumor stage	Surgical technique	Oncological outcomes	Main findings
Luo [9] et al.	T1	TLM vs. VPL	LC at 1, 3, 5 yrs: TLM (96.9%, 85.0%, 85.0%) vs. VPL (98.3%, 95.5%, 93.2%)	No statistically significant difference for T1; both techniques effective
Campo [4] et al.	T2	TLM vs. VPL	LC: VPL 94.4% vs. TLM 75.4%	VPL superior for advanced (T2) tumors
De Campora [5] et al.	T2	TLM vs. VPL	DFS: TLM 81.3% vs. VPL 85.7%	VPL may provide more durable oncological outcome
Mantsopoulos [10] et al.	Various	TLM vs. VPL	No significant difference in LC	Outcomes depend on proper patient selection
Karatzanis [11] et al.	Various	TLM vs. VPL	No significant difference in LC	TLM suitable for well-delineated lesions; VPL better for infiltrative tumors

*TLM* transoral laser microsurgery, *VPL* vertical partial laryngectomy, *LC* local control, *DFS* disease-free survival

### Recurrence rates and salvage surgery

The potential for local recurrence remains a critical concern, particularly for TLM. Several studies, including those by De Campora et al. [5] and Sewnaik et al. [12] have observed that TLM is associated with a higher recurrence rate, especially in cases involving the anterior commissure. Specifically, De Campora et al. [5] reported a recurrence rate of 17.8% for TLM compared to 12.2% for VPL. Sewnaik et al. [12] further demonstrated that in cases where the anterior commissure was affected, TLM resulted in recurrence in 55.5% of such cases, underscoring the limitations of this approach in tumors extending into this region​​.

Conversely, a study by Harada et al. [13] suggested that while TLM is associated with a higher risk of recurrence, it also allows for easier salvage surgery, potentially leading to better functional outcomes upon recurrence compared to VPL. These findings indicate that while OPL may provide better initial control, TLM offers enhanced flexibility in re-treatment, making it an appealing option for patients for whom organ preservation is a priority (Table 3).

**Table 3:** Recurrence rates after TLM or VPL

Study	Tumor characteristics	Surgical technique	Recurrence rate	Main findings	Laryngeal preservation rate
De Campora [5] et al.	General	TLM vs. VPL	TLM: 17.8% vs. VPL: 12.2%	Higher recurrence with TLM	93,6% in FLL, 91,9% in laser cordocommissurectomy
Sewnaik [12] et al.	Anterior commissure involvement	TLM	Recurrence in 55.5%	High recurrence with TLM in anterior commissure tumors	76.2% in FLL, 59.5% in TLM
Harada [13] et al.	General	TLM vs. VPL	Higher recurrence with TLM	TLM allows easier salvage surgery, better functional outcomes post-recurrence	100% after VPL, 80% after TLM

*TLM* transoral laser microsurgery, *VPL* vertical partial laryngectomy

### Postoperative morbidity and functional outcomes

Beyond oncological control, the impact of these surgical approaches on postoperative complications, voice quality, and overall patient quality of life (QoL) is of paramount importance (Table 4). Studies consistently indicate that patients undergoing VPL experience higher perioperative morbidity. Langer et al. [14] reported that patients who underwent VPL had significantly longer hospital stays, increased rates of tracheostomy, and prolonged dependency on nasogastric tube - factors that can substantially affect postoperative recovery and QoL. In contrast, TLM is generally associated with a faster recovery rate and fewer complications. However, a notable downside is the higher incidence of postoperative granulomas. De Campora et al. [5] found that 19.2% of patients treated with TLM developed granulomas, compared to 6.2% of those undergoing VPL, which may necessitate additional interventions to optimize voice outcomes​.

**Table 4:** Functional outcomes after VPL or TLM

Study	Surgical technique	Postoperative morbidity	Voice outcomes	Swallowing/QoL
Langer [14] et al.	VPL vs TLM	VPL: longer hospital stay, higher tracheostomy and NGT dependency	No significant difference long-term	n/a
De Campora [5] et al.	TLM vs VPL	TLM: granulomas in 19.2% vs OPL: 6.2%	Granulomas may affect voice	n/a
Harada [13] et al.	TLM vs VPL	n/a	n/a	TLM: better preserved swallowing, lower aspiration and dysphagia rates

Despite these challenges, long-term functional outcomes, particularly regarding vocal recovery, appear to be comparable between TLM and VPL. Langer et al. [14] found no significant difference in long-term voice recovery between the two approaches, suggesting that while VPL may initially have a more substantial impact on vocal function, patients ultimately regain similar levels of vocal performance​.

Furthermore, Harada et al. [13] highlighted that swallowing function was better preserved in patients undergoing TLM, with lower rates of aspiration and postoperative dysphagia. This reinforces the preference for TLM in patients prioritizing functional outcomes.

## Discussion

The management of early-stage glottic carcinoma requires a careful balance between achieving optimal oncological control and preserving laryngeal function. Two primary surgical approaches, TLM and VPL are commonly employed, each with distinct advantages and limitations. The results of this systematic review offer a nuanced comparison between these two techniques, emphasizing both oncologic efficacy and functional outcomes.

TLM has emerged as the preferred option for T1 glottic tumors, with studies showing comparable local control (LC) and disease-free survival (DFS) to VPL in well-selected cases. For instance, Luo et al. [9] demonstrated similar 5-year LC rates for TLM (85.0%) and VPL (93.2%) in T1 tumors, with no statistically significant difference. However, when addressing T2 lesions, especially those involving the anterior commissure, VPL showed superior oncologic performance. Campo et al. [4] reported a striking difference in LC (VPL: 94.4% vs. TLM: 75.4%), underscoring the potential benefit of VPL in more advanced or anatomically challenging cases.

This advantage in oncological outcomes, however, comes with trade-offs. Postoperative morbidity is generally higher in VPL, with increased rates of tracheostomy, longer hospital stays, and dependence on nasogastric tubes, as reported by Langer et al. [14]. Conversely, TLM is associated with faster recovery and fewer complications, albeit with a higher recurrence rate, particularly in tumors involving the anterior commissure [5, 12].

Interestingly, while VPL may reduce the need for total laryngectomy in the short term due to better local control, TLM allows for more flexible salvage strategies. Harada et al. [13] emphasized the ease of repeated transoral procedures and better post-recurrence functional outcomes with TLM, making it a more suitable option for patients where organ preservation is a key priority.

Another important aspect highlighted by this review is the rate of laryngeal preservation following treatment failure. While exact numbers vary, the need for salvage total laryngectomy after recurrence was slightly higher in VPL-treated patients due to more extensive resections and limited re-treatment options, whereas TLM-treated patients were more often candidates for conservative re-interventions [13].

VPL generally offers superior local control and lower recurrence rates, TLM allows for multiple conservative reinterventions, even in the setting of repeated recurrences. This therapeutic flexibility may explain the slightly lower laryngeal preservation rates reported in some studies for TLM, which often includes patients with more advanced or multiply treated disease at the time of salvage. Harada et al., for instance, reported a 100% laryngeal preservation rate after VPL, compared to 80% after TLM [13]. Similarly, lower preservation rates were noted for TLM in anterior commissure-involving tumors (59.5% in the study by Sewnaik et al.), yet this may reflect tumor complexity rather than inherent limitations of the technique [12]. These findings underscore the importance of interpreting preservation rates in the context of disease stage, recurrence burden, and the availability of repeated minimally invasive procedures.

TLM also provides several practical advantages that enhance its appeal as an upfront treatment for early glottic carcinoma. These include:

- Custom-tailored resections, allowing precise oncologic margins while minimizing tissue damage [15];
- Shorter hospitalization times and faster postoperative recovery, translating into reduced healthcare costs [16];
- Lower complication rates and reduced morbidity, especially regarding airway and swallowing outcomes [17];
- Favorable cost-effectiveness, with significant savings both per patient and institutionally in high-volume centers [18];
- No preclusion to further surgical or non-surgical salvage therapy, offering flexibility in long-term treatment planning [19].

Considering the results of this review, it may be reasonable to state that VPL can still be performed safely in selected cases. However, it is important to emphasize that trends in the use of this surgical technique vary significantly depending on geographical distribution and institutional capabilities. In particular, there is a clear divergence between Europe and South America. VPLs are still commonly performed in South America, where TLM is less frequently adopted. In contrast, in Europe, VPLs have been largely replaced by TLM and open partial horizontal laryngectomies (OPHL), reflecting broader availability of equipment and greater expertise in minimally invasive approaches.

These differences are influenced by contextual and epidemiological factors. In Europe, laryngeal cancer is often diagnosed at earlier stages, allowing for conservative treatments. In South America, late-stage diagnoses are more frequent, necessitating open surgical approaches such as VPL [20]. Additionally, epidemiological trends differ: while the incidence of laryngeal cancer has been decreasing in Europe due to effective public health policies on alcohol and tobacco, it remains on the rise in several South American regions where these risk factors are still prevalent [20].

These contextual differences underscore the importance of institutional capabilities and public health infrastructure in shaping surgical choices. Beyond clinical indications, the availability of laser technology, surgeon experience, and economic considerations play pivotal roles in determining the optimal treatment approach.

Finally, it is important to recognize the limitations of this systematic review. Variability in study design, patient selection, and outcome reporting across the included studies may introduce bias. The findings should therefore be interpreted with caution, and future prospective, multi-institutional studies are needed to validate these observations and guide treatment algorithms.

Additionally, the possibility of publication bias must be considered, as studies reporting positive or significant findings are more likely to be published. Furthermore, notable heterogeneity was observed among the included studies in terms of patient selection, tumor staging, surgical techniques, and outcome reporting. These variations could affect the generalizability and comparability of the findings. Future studies should aim to use standardized outcome measures and prospective designs to reduce such heterogeneity and improve data consistency.

## Conclusion

Both TLM and VPL are effective surgical strategies for early-stage glottic carcinoma, each with distinct advantages and limitations. TLM offers the benefits of minimally invasive surgery, including improved functional outcomes and the potential for repeated interventions in the event of recurrence. Conversely, VPL may be more suitable in cases with challenging tumor characteristics. Ultimately, the choice of treatment should be individualized based on tumor characteristics, patient preferences, and institutional expertise to achieve the best oncological and functional outcomes.

## Data Availability

The data used in this study are available upon request from the corresponding author.
